# Measuring Dynamic and Kinetic Information in the Previously Inaccessible Supra-τ_c_ Window of Nanoseconds to Microseconds by Solution NMR Spectroscopy

**DOI:** 10.3390/molecules181011904

**Published:** 2013-09-26

**Authors:** David Ban, T. Michael Sabo, Christian Griesinger, Donghan Lee

**Affiliations:** Department for NMR-based Structural Biology, Max-Planck Institute for Biophysical Chemistry, Am Fassberg 11, D-37077 Göttingen, Germany

**Keywords:** supra-τ_c_, RDC, relaxation dispersion, R_1ρ_, CPMG, protein dynamics, NMR spectroscopy

## Abstract

Nuclear Magnetic Resonance (NMR) spectroscopy is a powerful tool that has enabled experimentalists to characterize molecular dynamics and kinetics spanning a wide range of time-scales from picoseconds to days. This review focuses on addressing the previously inaccessible supra-τ_c_ window (defined as τ_c_ < supra-τ_c_ < 40 μs; in which τ_c_ is the overall tumbling time of a molecule) from the perspective of local inter-nuclear vector dynamics extracted from residual dipolar couplings (RDCs) and from the perspective of conformational exchange captured by relaxation dispersion measurements (RD). The goal of the first section is to present a detailed analysis of how to extract protein dynamics encoded in RDCs and how to relate this information to protein functionality within the previously inaccessible supra-τ_c_ window. In the second section, the current state of the art for RD is analyzed, as well as the considerable progress toward pushing the sensitivity of RD further into the supra-τ_c_ scale by up to a factor of two (motion up to 25 μs). From the data obtained with these techniques and methodology, the importance of the supra-τ_c_ scale for protein function and molecular recognition is becoming increasingly clearer as the connection between motion on the supra-τ_c_ scale and protein functionality from the experimental side is further strengthened with results from molecular dynamics simulations.

## 1. Introduction

One of the essential keystones for the existence of life rests in the intricate relationship between biomolecular function and structural dynamics. The biomolecular machines engaging in these indispensable processes possess internal structural dynamics on a wide range of time-scales. It is the relationship or connection between the time-scales of these fundamental biophysical phenomena and the time-scales of biomolecular dynamics that the technique of Nuclear Magnetic Resonance (NMR) spectroscopy is uniquely equipped to explore. NMR is a powerful technique whose observables are time-scale sensitive [1]. Given that the sample is tractable for NMR studies, the system can be explored in solution without chemical modification while maintaining atomic resolution. 

**Figure 1:**
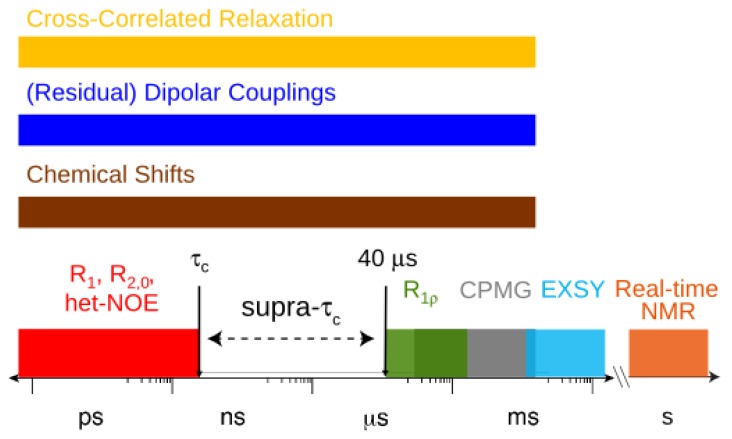
The accessible time-scale for NMR observables and the respective limits for some NMR experiments.

A wide range of NMR experiments have been developed that report on a broad range of time-scales from picoseconds to more than seconds (Figure 1). Exchange Spectroscopy (Figure 1; EXSY) first demonstrated by Meier *et al.* in 1979 [2] is a NMR technique for investigating slow time-scale dynamics (~50 ms to more than seconds provided that the exchange process is not much slower than the longitudinal relaxation rate). Specifically slow processes can therefore be studied by EXSY on states of the density matrix with long longitudinal relaxation times [2]. EXSY was later applied to measure aromatic ring flips in BPTI [3], to monitor enzyme catalysis [4,5,6] and to follow folding processes [7,8]. NMR can also be implemented for the investigation of biophysical processes occurring so slowly that several free induction decays (FIDs) or even multidimensional spectra can be recorded while the system is pseudo-static. This type of experiment is called real-time NMR and utilizes the repetitious collection of NMR spectra. Real-time NMR has been applied to systems engaged in slow substrate turn-over events [9] and in folding processes [10]. Fast acquisition techniques [11,12,13] and hyperpolarization [14,15] have recently been invented with the goal of measuring faster kinetics as well as improving signal to noise. Additionally, the probed system adopts a range of distinctive structural configurations that may translate into differences in the NMR chemical shift. Depending on the time-scale for the structural changes, the chemical shift itself can also be used as a dynamic metric [16,17,18,19]. Chemical shifts have been predicted and used to report on sampled conformational sub-states from a recent 1 millisecond molecular dynamics (MD) trajectory of bovine pancreatic trypsin inhibitor (BPTI) [19,20]. Another important NMR parameter is the cross-correlated relaxation rate, which encapsulates the correlated nature of potentially synchronous inter-nuclear vector motion on time-scales spanning picoseconds to milliseconds [21,22,23,24,25].

In this review, we address the considerable progress that has been made to characterize the amplitude and time-scale of inter-nuclear vector dynamics within a window that remained as a “blind-spot” for NMR. This window covers about four orders of magnitude and ranges between the overall tumbling time of a molecule (τ_c_) to 40 μs (Figure 1), but as we will see has been extended to ~25 μs [26] (Section 3). This previously inaccessible time window has been defined as the supra-τ_c_ range [27,28]. The use of experiments that report on motions faster than τ_c_ will also be mentioned as well as the experiments denoted in Figure 1 as rotating frame longitudinal relaxation (R_1ρ_) and Carr-Purcell-Meiboom-Gill (CPMG) experiments, which rely on the characterization of conformational exchange. The extraction of dynamical content from residual dipolar couplings (RDCs) and the measuring of kinetics from conformational exchange has implications for understanding the mechanisms of molecular recognition and protein function within the supra-τ_c_ scale.

## 2. Dynamic Content of the Supra-τ_c_ Range

### 2.1. Introduction to Residual Dipolar Couplings

An essential NMR parameter that provides both structural and dynamic information including the supra-τ_c_ scale is the residual dipolar coupling (RDC) between two nuclear magnetic moments [29,30]. The beginnings of the RDC field can be traced back to 1963 when Saupe and Englert aligned benzene with *p*-azoxyanisole [31]. From here, the theoretical background for the analysis of RDCs for the extraction of structural and dynamic parameters has a rich history of development. Though this review does not aim to provide a detailed account concerning the history of the RDC field, nevertheless, we refer the reader to a comprehensive, yet not exhaustive, list of the pioneering work from Saupe [32,33], Snyder [34], MacLean and co-workers [35,36,37], Emsley and co-workers [38,39,40,41,42,43,44], and Pines and co-workers [45,46] toward the advancement of the theoretical underpinnings regarding solute alignment under anisotropic conditions.

In respect to biomolecules, thirty-two years passed from the initial report from Saupe and Englert [17] until Tolman and coworkers reported the first investigation of aligning cyanometmyoglobin, which possesses a paramagnetic center, in a magnetic field [47]. At the same time, Bolton and co-workers aligned a dodecamer of DNA in a magnetic field due to magnetic susceptibility of the unlabeled DNA [48]. A year later, Bax and colleagues described RDCs for the diamagnetic protein ubiquitin taken from changes in N-H^N^ splitting resulting from varying magnetic susceptibility anisotropy at different static field strengths [49]. These couplings were quite small, on the order of 0.2 Hz, and an alternative method for measuring RDCs was proposed where the protein is dissolved in a partially anisotropic environment, the first being bicelles [9]. Since then, a multitude of alignment media have been described in the literature, including but not limited to filamentous phages [50,51,52], a mixture of cetylpyridinium bromide/chloride hexanol [53,54], a mixture of alkyl poly(ethylene glycol) and hexanol [55], a stretched [56,57] or a compressed polyacrylamide gel [58], and purple membrane fragments [59,60]. The advantage of these types of alignment media is that the strength of alignment is more under the experimentalist’s control, producing RDC values several orders of magnitude higher than in the case of magnetic susceptibility anisotropy. For more information on the specifics of the different types of alignment media that are currently available, the reader is referred to the following reviews [61,62,63].

In the anisotropic media, all possible orientations for an inter-nuclear vector are populated with unequal probability, resulting in the dipolar couplings (*D*) no longer averaging to zero, with values on the order of 1/1000 the value of the maximal dipolar coupling. From an experimental standpoint, the RDC adds together with *J*-coupling, requiring two measurements to extract the RDCs: one in isotropic conditions to determine the *J*-coupling and one in anisotropic conditions to determine the (*J* + *D*)-coupling. Reviewed in several places, schemes for measuring the RDCs either rely on measuring the peak splitting in a coupled HSQC or the (*J* + *D*)- or *J*- coupling modulated peak intensity [61,64,65,66].

The main focus of this section is two-fold. First, a theoretical framework for utilizing RDCs in the determination of structural and dynamic properties for inter-nuclear vectors is described in detail. The measured RDCs encode a unique signature of the extent of supra-τ_c_ motion and methodology has been developed toward extracting this information in a robust manner. The second half of this section concentrates on some of the effort that has been invested in linking these amplitudes of supra-τ_c_ motion to biophysical phenomena, specifically molecular recognition. In particular, long MD trajectories, accelerated molecular dynamics simulations, and RDC restrained molecular dynamics simulations provide a more global picture of how these experimentally derived local dynamics could be relevant for function and stability.

### 2.2. Alignment Tensor Determination

Partial alignment of a protein results in the observed resonance splitting (Hz) for two nuclear spins to possess a contribution from the secular part of the magnetic dipole interaction:

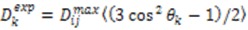
(1)

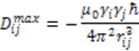
(2)
where *μ*_0_ is the permeability of vacuum, *γ_i_* and *γ_j_* are the gyromagnetic ratios of nuclei *i* and *j*, ℏ is Planck’s constant, *r_ij_* is the distance between nuclei *i* and *j*, and *θ_k_* is the angle between the inter-nuclear vector formed by nuclear spin pair *k* and the magnetic field (*B*_0_). The important concept to note from Equation (1) is that the magnitude of 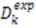 depends on 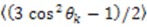, which is ensemble averaged over the time-scale covered by the RDC measurement (denoted by the angular brackets). The time-averaged or ensemble averaged information covers up to the millisecond time-scale or roughly 1/*D*, spanning the supra-τ_c_ scale [30].

We will first consider the simplest case, theoretical analysis of a rigid molecule. In the absence of inter-nuclear vector dynamics, the instantaneous orientation of *B*_0_ relative to each inter-nuclear vector within a protein or molecule can be defined in the molecular frame (MF), which is an arbitrary frame of reference usually given by the PDB coordinates. Each inter-nuclear vector can be defined by three angles, *β_x_*, *β_y_*, and *β_z_*, between the vector and the respective MF axes. In a similar fashion, the vector parallel to *B*_0_ can be expressed by three angles representing the instantaneous orientation of *B*_0_ relative to the MF axes, *α_x_*, *α_y_*, and *α_z_*. Within the MF, Equation (1) can be expressed as:

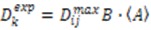
(3)
where *B* ∙ 〈*A*〉 is the scalar product of two vectors representing the inter-nuclear orientations (*B*) and the *B*_0_ orientations (〈*A*〉). Both 〈*A*〉 and *B* contain 5 independent terms and are related to a 3 × 3 second rank Cartesian order tensor as follows [32,33,34]

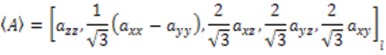
(4)
where the averaged orientation of *B*_0_ in the MF is given by:

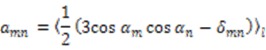
(5)
and:

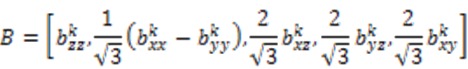
(6)
where the orientation of the inter-nuclear vector in the MF is described by:

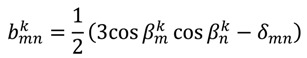
(7)

The term *δ_mn_* represents the Kronecker delta function, *l* is the alignment condition, and *m*,*n* = *x*, *y*, *z*. When a set of RDCs have been measured for a protein and the structural coordinates of the protein are known from a crystal or NMR structure, then Singular Value Decomposition (SVD) is typically used to calculate an exact solution for the alignment tensor, 〈*A*〉** [67]**. Here, SVD of *B* is performed in order to obtain the pseudo-inverse of *B*, *B*^+^. With *B*^+^, the following equation determines 〈*A*〉 through left multiplication of Equation (3) with *B*^+^:

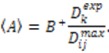
(8)

The SVD approach requires that the RDC measurement includes at least five inter-nuclear vectors sampling at least five independent orientations, leading to a nonsingular *B* matrix and thus *B^+^ B = 1*, or the identity matrix.

The calculated alignment tensor can be recast into a symmetric 3 x 3 second rank Cartesian order tensor, (〈*A*^(2)^〉) and then redefined in a principal axis system (PAS), termed the alignment frame (AF), where Equation (1) becomes [61]:

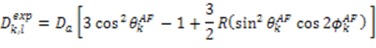
(9)

In Equation (9), the magnitude of the alignment tensor is 
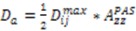
, the rhombicity is 
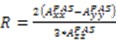
, 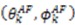 are the polar angles defining the inter-nuclear vector in the AF, and 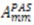 are the eigenvalues resulting from the diagonalization of 〈*A*^(2)^〉. From the eigenvectors (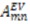), the Euler angles describing the rotation of 〈*A*〉 and *B* into the PAS are defined:

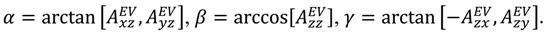
(10)

In the case where multiple RDC sets are available for a given biomolecule, Joel Tolman developed a compact matrix formalism to calculate alignment tensor information in a more intuitive manner [68]. When *K* RDCs are measured under *L* alignments, then Equation (3) becomes:

**D** = **B**〈**A**〉 (11)

where **D** is a *K* x *L* matrix, **B** is a *K* x 5 matrix, and 〈**A**〉 is a 5 x *L* matrix. In Equation (11), the term 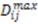 is included in 〈**A**〉. The rows of **B** are defined by Equation (6) and the columns of **A** are given by Equation (4). As with the SVD approach to a single alignment media, the calculation of 〈**A**〉 for all alignment media at once requires a nonsingular **B** matrix:

〈**A**〉 = **B**^+^**D** (12)

where **B**^+^ is the pseudo-inverse of **B**. As mentioned above, each alignment tensor can be rotated into a PAS and the five parameters describing the alignment, {*D_a_, R, α, β, γ*}*_l_*, can be extracted with Equations (9) and (10). It should be mentioned that several programs exist for the calculation of alignment tensor information, such as PALES [69,70] and REDCAT [71].

### 2.3. Model Free Analysis and Direct Interpretation of Dipolar Couplings

In reality, the molecules under investigation are not static and ensemble averaging of the RDC observable must also be considered. To calculate the dynamical content from the RDC data, five orthogonal alignment media must be measured for the system under investigation. This requirement is in analogy to the determination of an alignment tensor, where at least five orthogonal inter-nuclear vectors are necessary to define the five independent elements of each alignment tensor. The dynamical content contained within the RDC data is encapsulated by a generalized order parameter, 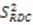, which possesses dynamic information on the picosecond to millisecond time-scale, which includes the supra-τ_c_ range (Figure 1). Within the framework of ensemble averaging, Equations (11) and (12) become:

**D** = 〈**B**〉〈**A**〉 (13)

〈**A**〉 = 〈**B**〉^+^**D** (14)

There are two principal schemes for extracting the structural and dynamic content from RDC data measured in at least five linearly independent alignment media, namely the Model Free Analysis (MFA) [72] and the Direct Interpretation of Dipolar Couplings (DIDC) [68]. Before delving into the details concerning these model free approaches, we discuss a necessary caveat for the implementation of this methodology and how to assess the quality of the RDC data, as well as the sampling of the five-dimensional space.

The fundamental assumption for both the MFA and DIDC approaches is that the internal protein dynamics for each inter-nuclear vector is uncorrelated with alignment tensor. Thus, a single average alignment tensor can be utilized for each medium. Molecular dynamics simulations indicate that this assumption is true for secondary structural elements, yet 〈**B**〉 and 〈**A**〉 dynamics may be correlated for the most mobile regions of a protein [73,74]. In the case of intrinsically disordered proteins [75,76], multi-domain proteins [77,78], and extended nucleic acid structures [48,79], internal conformational dynamics couples with the alignment tensor. After realizing that even intrinsically disordered proteins exhibit RDCs [80,81], Blackledge and co-workers have developed strategies for interpreting RDCs measured for intrinsically disordered proteins and the reader is referred to the following papers on the subject [82,83,84]. RDCs measured for multi-domain proteins report on both inter-nuclear vector dynamics and inter-domain dynamics that result in changes in molecular alignment [85]. One avenue to de-correlate these two processes is to use internal paramagnetic alignment where one domain is preferentially aligned in the magnetic field [78,86]. Though the focus of this section is assessing inter-nuclear vector dynamics via RDCs obtained from external alignment media, we direct the interested reader to these papers on utilizing paramagnetic tagging for the determination of structure, inter-domain orientation and dynamics with RDCs from the groups of Ubbink [87,88], Bertini [78,89,90], Otting [91,92], Byrd [93,94], and Schwalbe [95,96]. As for aligning extended nucleic acid structures, such as RNA, the laboratory of Al-Hashimi has developed a novel strategy of elongating RNA helices [97]. In this situation, the extended RNA structure dominates the molecular alignment and the internal dynamics of the RNA helix no longer contributes to the overall alignment of the biomolecule [98].

When analyzing RDC data, a fundamental assumption that is made concerns the uncorrelatedness of the inter-nuclear dynamics and the alignment process; hence the averages of 〈**B**〉 and 〈**A**〉 are independent of each other. This assumption can be tested with the Self-Consistency of Dipolar Couplings Analysis (SECONDA) [99,100]. SECONDA attempts to quantitate the degree of structural heterogeneity for the measured protein over at least 6 different alignment media. The analysis only requires RDC data as input, without structural data or alignment tensor information. In principal, perfectly homogenous behavior suggests that the internal structural dynamics are not influenced by the variations in the alignment process, in temperature or in pH. Furthermore, the homogeneity can be quantified on a per residue basis. A covariance matrix is created from the RDC data and from a principal component analysis or SVD of the covariance matrix the degree of structural heterogeneity is assessed from the resulting singular values. The first five singular values contain the structural and dynamic content encompassed within the RDC data. The other singular values indicate the degree of structural and dynamic heterogeneity, noise, and systematic errors resulting in singular values that are not equal to zero. The SECONDA gap is a measure of the self-consistency and homogeneity of the RDC data set, which is the ratio of the 5th and 6th singular value.

The degree in which the five dimensional space is being sampled can be quantified from SVD [68,101]. SVD of the five independent elements of each alignment tensor within 〈**A**〉 yields five singular values. The ratio of the first to the fifth singular value indicates the strength of the contribution from the strongest and weakest dimensions being sampled by the RDC data. This parameter is called the condition number. A value of one indicates that each dimension is being sampled equally. In the case of ubiquitin where 36 RDC data sets were available the average condition number was 6.3 [102]. Residues 22, 31, 46, 69, and 73 were removed from this 36 RDC data set, since they possessed condition numbers larger than 10.

We will focus our discussion on the extraction of structural and dynamic content from RDC data on the similar nature of both the MFA and DIDC analysis and how they are unified within the context of the standard tensorial analysis [103]. Beginning with the MFA, five independent alignment media are necessary to calculate the five independent elements of the RDC tensor for each inter-nuclear vector, as well as *a priori* knowledge of the protein structure [72,101]. With the alignment tensor information, the averages over the second rank spherical harmonics describing the mean orientations of the vectors, contained within the RDC tensor, provide the desired structural and dynamic content. The alignment tensor parameters taken from the alignment frame (AF), {*D_a_, R, α, β, γ*}*_l_*, are used to construct the 〈**F**〉 matrix which is needed to derive the five dynamically averaged second order spherical harmonics:

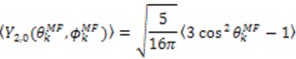
(15)

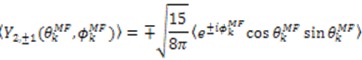
(16)

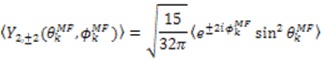
(17)

Utilizing the following relationships,

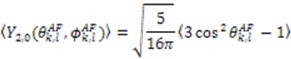
(18)

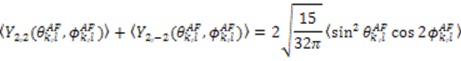
(19)

Equation (9) is reformulated in terms of dynamically averaged second order spherical harmonics

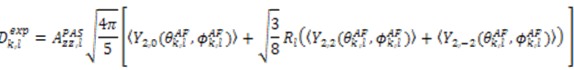
(20)

The 〈**F**〉 matrix functions in a similar way to 〈**A**〉 and relates the measured RDCs to the spherical harmonics defined in the MF by a Wigner rotation from the MF to the AF:

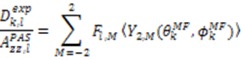
(21)
with:

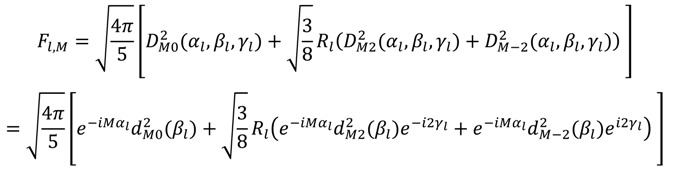
(22)

In analogy to the component definition from Equations (13) and (14), 〈**Y**〉 is a *K* x 5 matrix containing the dynamically averaged spherical harmonics in the MF and 〈**F**〉 is a 5 x *L* matrix containing the alignment tensor information. Refined structural coordinates are determined directly from the RDCs and alignment information:

〈**Y**〉**_refined_** = **D_normalized_**〈**F**〉^+^ (23)

In order to normalize the contributions of each alignment condition to the calculation of refined structural coordinates, **D_normalized_** represents 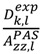 which results in the condition number being lower than in the unnormalized case. In other words, this normalization helps to even out the contributions of each RDC set to the calculation of **〈Y〉_refined_.** Each row of **〈Y〉_refined_** is used to determine 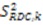:

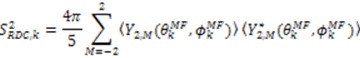
(24)

From the dynamically averaged spherical harmonics, the dynamically averaged orientations for each inter-nuclear vector, 
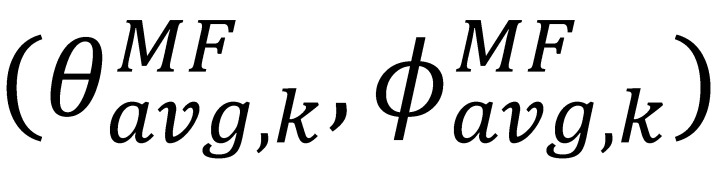
, can be obtained. Maximizing 
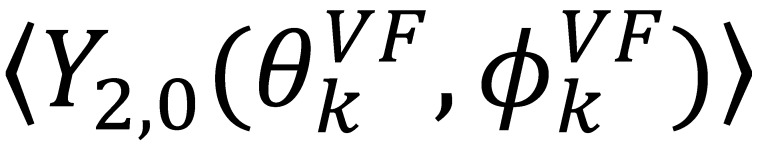
 places the z-axis of the vector’s axis system, termed the vector frame (VF), in the center of the inter-nuclear vector’s orientational distribution:

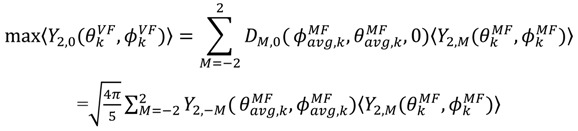
(25)

The terms 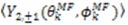 vanish in the VF and 
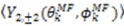
 possesses information on the amplitude of anisotropy, *η_k_*, and the orientation of anisotropic motions, 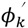:

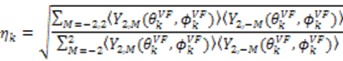
(26)

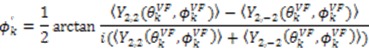
(27)

It should be noted that 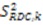 is the same in any frame, thus:

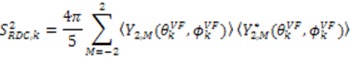
(28)
which is equivalent to Equation (24).

With the DIDC approach, structural input is not required in the calculation of the inter-nuclear vector’s structural and dynamic content [68]. In concordance with Equation (23):

〈**B**〉**_refined_** = **D**〈**A**〉^+^ + **B**[**1** – 〈A〉〈A>〉^+^] (29)

where 〈**B**〉**_refined_** can be calculatedwithout extracting each set of {*D_a_, R, α, β, γ*}*_l_*. A key difference between the MFA and DIDC is the requirement of normalizing **D** and 〈**A**〉. In the current implementation of DIDC, these parameters are not normalized, which will lead to some discrepancies between RDC analysis utilizing the MFA and the DIDC. Neglecting this normalization will lead to a disproportionate contribution of the stronger alignment media to the calculation of the refined structural coordinates. We propose the following modification of Equation (29) for the DIDC approach when seeking a direct correspondence between both methods:

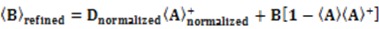
(30)
where **D_normalized_** and 〈**A**〉**_normalized_** represent the RDCs and alignment tensors divided by 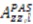.

The unification of both the MFA and the DIDC is readily apparent when looking at the relationship between 〈**B**〉 and 〈**Y**〉. Recalling Equation (6), the following relationships are established in order to construct 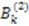 [34]:

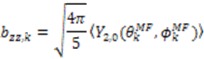
(31)

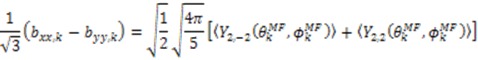
(32)

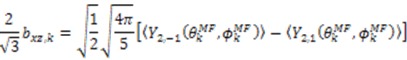
(33)

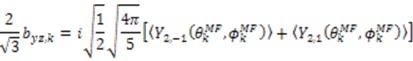
(34)

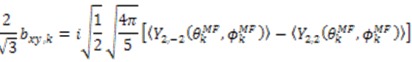
(35)

In 2012, Meirovitch *et al.* made the following connection between maximizing 
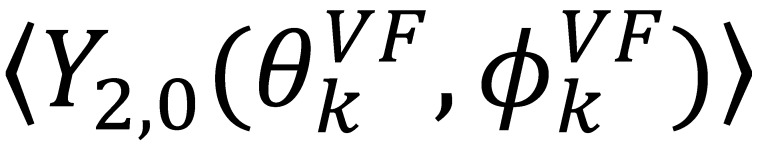
 and defining each inter-nuclear vector in a unique principal axis system [103]. The resulting eigenvalues (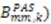 contain the dynamic information for each vector 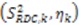, while the eigenvectors, 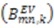, encompass the bond orientations 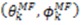 and the direction of the anisotropic local motion 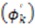. The following equations detail how the dynamic parameters are calculated from 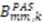. The Saupe order parameters are defined as:

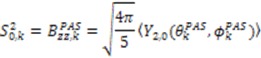
(36)

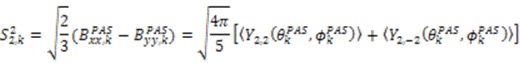
(37)

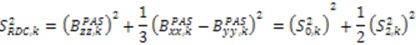
(38)

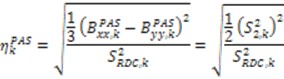
(39)

For each inter-nuclear vector, 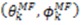 and 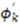 are extracted from the transpose of the resulting 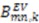 matrix:

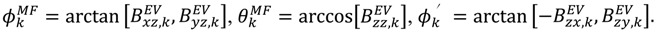
(40)

Both the MFA and DIDC methods have been incorporated into iterative schemes with the goal of improving the accuracy of the alignment tensor calculation by reducing the effects of the structural noise, termed the Self-Consistent RDC based MFA (SCRM) [102], iterative DIDC [104] and the Optimized RDC-based Iterative and Unified Model-free analysis (ORIUM) [89]. The iterative schemes achieve this by using the refined dynamically averaged coordinates as input for additional runs of either MFA or DIDC. Interestingly, ORIUM is the only iterative procedure that can begin with random coil input as the starting structural coordinates and extract the same structural and dynamic information calculated from an x-ray structure used as the beginning structural input [105]. This result shows that the iterative ORIUM scheme tolerates a significant amount of structural noise and has the potential be implemented in refinement of conformational ensembles.

A final consideration when determining the dynamic parameters from RDC data is that the actual magnitude of 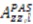 or *D _a,l_* is not known, which will lead to 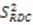 values being only relative in nature to the true absolute value [27,72]. The other alignment tensor parameters {*R, α, β, γ*}*_l_* are unaffected by the reduction in the magnitude of *D _a,l_*. The correct scaling parameter, termed *S_overall_*, is crucial for distinguishing sub- and supra-τ_c_ motion. All three iterative schemes have addressed this issue in different manners. In the iterative DIDC, order parameters are scaled relative to the largest 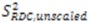 leaving one order parameter equal to one [68,104]. Sub- and supra-t_c_ motion happening for each vector equally will not be detected by this approach, which will underestimate the motion. With the MFA/SCRM procedure, 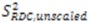 is scaled relative to the Lipari-Szabo order parameters (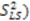 calculated for each residue [27,102], as long as 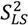 are available for the inter-nuclear vectors being analyzed. This approach as been successfully applied to ubiquitin, however, supra-t_c_ motion affecting all nuclei equally will not be picked up by this method. Finally, ORIUM uses the inter-nuclear vector’s motional variance, which is directly related to the resulting eigenvalues calculated from diagonalization of 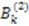 into a local axis system. By definition, variance cannot be negative, and therefore, a uniform scaling parameter, *S_overall_*, is necessary to insure that the variance for each inter-nuclear vector about each of the three principal axes is positive. The advantage of this method is that *S_overall_* is derived based on variances of a single type of RDC without needing 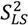 as a constraint, and hence does not possess any time-scale bias. Yet, it should be noted that *S_overall_* determined by this procedure could underestimate motion if there is a uniform sub- or supra-t_c_ motion affecting all inter-nuclear vectors equally.

### 2.4. Gaussian Axial Fluctuation Model

Brüschweiler and co-workers developed a model, termed the Gaussian Axial Fluctuation model (GAF), describing rotational motion of the peptide plane around the axis defined by Cα(*i*-1) and Cα(*i*) [106,107,108]. The original observation for this type of motion comes from NMR spin relaxation measurements as well as molecular dynamics simulations, where crankshaft type motions along this axis were observed. Blackledge and co-workers have adapted this model for the use with RDC data, which spans up to the millisecond time-scale [109,110,111]. The amount of this motion is encapsulated in the angular standard deviation (σ) about three orthogonal axes defined by the peptide plane. In the simplest formulation, considering motion about the axis defined by Cα(*i*-1) and Cα(*i*), the model is considered (ortho-GAF) and σ is incorporated into Equation (6) as follows:

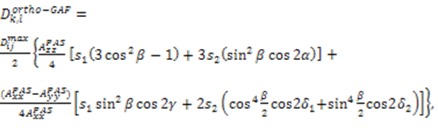
(41)
where:

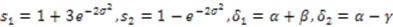
.
(41)

Here, *α, β, γ* represent the Euler angles describing the transformation into the peptide plane’s frame, where the z-axis points along the direction of the N-H^N^ bond and the x-axis orthogonal to the peptide plane. This rotation is similar to putting the N-H^N^ bond in the local principal axis system described above. It should be noted that the Cα(*i*-1) and Cα(*i*) axis is tilted away from the y axis by 11 degrees.

As is this case with RDCs measured in alignment media with unknown absolute magnitude, the GAF method also requires a scaling factor, which has been addressed with the structure-free (SF) GAF approach [112]. The methodology treats each peptide plane as an independent entity, the only requirement being the starting coordinates for a representative peptide plane. RDC data measured in multiple alignment media for the N-H^N^, C'N, C'- H^N^, and C'-Cα were used to fit for the residue specific *α, β, γ, σ_z_, σ_y_ ,σ_x_* and the alignment condition specific 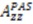. This procedure was most effective when using the full 3D-GAF model. For ubiquitin, a comparison of 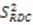 derived from the SCRM [102] approach of constraining with 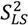 with the SF-GAF method 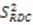 displayed remarkable agreement.

### 2.5. Supra-τ_c_ Dynamics Determined from RDCs is linked to Molecular Recognition

The potential linkage of experimentally extracted inter-nuclear vector dynamics within the supra-τ_c_ range to molecular recognition has been investigated for three proteins: ubiquitin (SCRM [102]/SF-GAF [54]/ORIUM [105]), GB3 (iterative DIDC [45]/3D-GAF [111], ORIUM [105]) and SH3 (3D-GAF [113]). Amplitudes of supra-τ_c_ motion can be readily quantified from knowledge of 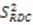 and 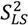:

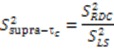
(42)

For all three proteins, additional motions resulting from slower time-scale dynamics are present. Furthermore, in the case of ubiquitin, there exists a significant amount of motion for the side-chain methyl groups [114], while such studies have not yet been conducted for the other two proteins. The connection to molecular recognition rests in the observation that for some residues possessing significant 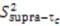 are involved with binding to interaction partners. When considering the interacting partners SH3 and ubiquitin, they both appear to possess a significant amount of supra-τ_c_ motion at their respective binding interfaces [102,113].

Molecular dynamics (MD) simulations restrained with or validated against RDC data is an approach to develop an ensemble of structures that best represents the ground state ensemble of the protein. Recently, RDC data was used as a restraint in MD simulations of ubiquitin. A principal component analysis of the resulting ensemble demonstrated that ubiquitin samples the same conformational space as all conformers captured to date in crystal structures of ubiquitin complexes [28]. These findings support the concept of conformational selection, specifically that amplitudes of motion or dynamics resulting from conformational inter-conversion on the supra-τ_c_ scale are limiting the on-rates for complex formation [115]. For ubiquitin, a majority of these conformational dynamics are concentrated in a single collective mode described by a pincer-like motion. To alleviate the entropic costs associated with sampling conformers along the pincer-like trajectory, another ubiquitin conformational ensemble predicts a significant amount of correlated motions, determined from long range ϕ/ψ dihedral correlations [116]. These findings demonstrate the power of using RDC data to refine ensembles containing dynamic information on the supra-τ_c_ scale.

Accelerated molecular dynamics (AMD) is another strategy for utilizing RDC data in the generation of a conformational ensemble possessing dynamics up to the millisecond time-scale [117]. In AMD, the energy barriers between the many conformational states of a protein ensemble are lowered, allowing the AMD simulation to cover conformational space that potentially exists on longer time-scales. From here, a canonical ensemble can be generated from a Boltzmann reweighting of each ensemble member. The AMD approach has been employed with several protein systems, including GB3 [118], ubiquitin [119], thrombin [120,121], and IκBα [122]. In all cases, the AMD ensembles were cross-validated with RDC data, then order parameters were calculated. Supra-τ_c_ motion is also present with the AMD method, suggesting, at least for systems as large as thrombin and IκBα, a common time-scale of supra-τ_c_ motion may be present for all proteins.

As this section has demonstrated, RDC data encompasses important information regarding the amplitudes of motion spanning the supra-τ_c_ scale. The next step is to assign a specific rate to this motion. Are these dynamics related to conformational inter-conversion within the ground state ensemble and, if so, what is the time-scale of this process? A powerful technique to potentially answer this question comes from relaxation dispersion measurements. The next section reviews the progress that has been made linking RDC extracted supra-τ_c_ scale dynamics to a specific rate describing the actual process of conformational inter-conversion.

## 3. Kinetics from the Supra-τ_c_ Range

### 3.1. Sub-τ_c_ Relaxation is Limited to the Overall Tumbling Time

Conventional NMR relaxation focuses on backbone ^15^N nuclei and we will limit our discussion to this nucleus type [123,124], although, a plethora of experiments have been developed that can probe the relaxation processes of other nuclei [125,126,127,128,129,130]. Commonly applied NMR experiments measure the longitudinal and transverse relaxation rates, R_1_ and R_2,0_, respectively [124]. These intrinsic relaxation parameters report on local oscillating magnetic fields that are generated due to dipolar interactions between the ^15^N nucleus and its attached amide proton (dipole-dipole coupling) and from magnetic fields generated due to a nucleus electron cloud’s orientation with the static magnetic field (chemical shift anisotropy) [131]. These local magnetic fields are reoriented because of molecular tumbling which for proteins occurs in the nanosecond range and demarcated by the characteristic lifetime called the overall rotational correlation time (τ_c_). These relaxation rates are described by spectral density type functions that are evaluated at characteristic frequencies, which report on transitions that drive nuclei back to equilibrium. These frequencies occur at zero, *ω*_H/N_, and *ω*_H_ ± *ω*_N_ where *ω*_X_ is the Larmor frequency for either ^1^H or ^15^N nuclei [131,132,133]. However, since these relaxation rates depend on fluctuations of dipolar couplings that are averaged by the molecular reorientation occurring with the correlation time τ_c_, only motions up to that time are accessible, *i.e.* motions that are slower than the inverse correlation time are not reflected in these relaxation rates. This range of motion faster than the inverse correlation time is called the sub-τ_c_ window. The experiments that probe these relaxation rates function by probing inphase coherences of the type N_x,y_ and N_z_ for R_2,0_ and R_1_, respectively [1,134], from which the longevity of these coherences is queried by observing their rate of decay which is extracted using a two parameter exponential decay function [135].

A broad range of information can be retrieved from the measurement of relaxation rates. The ratio of R_2,0_ and R_1_ can be used to determine τ_c_ itself [124], and many insights into local molecular flexibility can be attained by performing a Model-Free analysis (MF) [136,137]. The MF formalism allows the extraction of an order parameter that quantifies the spatial flexibility of a given inter-nuclear vector. This parameter can also be used as a proxy for conformational entropy [138,139,140,141]. An extended MF formalism has been developed for the characterization of internal motion occurring from two distinct time-scales within the sub-τ_c_ range [142]. The local and overall rotational anisotropy [143] of a macromolecule and the orientations of modular proteins [144,145] can also be ascertained from such data. Although insight into motion from the sub-τ_c_ range has given input into finer molecular motions, some biologically relevant processes cannot be assessed with these types of measurements due to the time-scale limitation.

The kinetic characterization for biologically relevant processes like protein folding [146,147,148,149], enzymatic turnover events [150,151,152,153,154], and molecular recognition [155,156,157] are inaccessible by conventional sub-τ_c_ relaxation techniques. Instead, NMR based relaxation dispersion (RD) experiments have emerged as a successful tool to explore these processes [1,158,159,160]. Fifty years ago, the concept of RD was applied for the measurement of the proton transfer rate between trimethylammonium and trimethylamine [161]. The success of these experiments is directly related to their ability to exploit the phenomenon of conformational exchange. Conformational exchange occurs when the electronic environment of a nucleus is perturbed due to its own motion or from its surroundings. This motion in turn modulates a nuclei’s isotropic chemical shift (*ω*) thereby causing the generation of alternatively populated coherences [159,162]. These variously populated states also interconvert with a given lifetime (τ_ex_) whose smallest observable value is limited by the experimental conditions. The effect of conformational exchange creates a dephasing in the transverse plane that is appended to R_2,0_. This dephasing creates an effective transverse relaxation rate, R_2,eff_ = R_2,0_ + R_ex_ where R_ex_ is a contribution of relaxation due to conformational exchange.

The lifetime for a conformational exchange event is governed by the chemical shift time-scale [163]. Traditionally, the process is defined to be either in the slow, intermediate, or fast regime. These regimes are separated based on the inverse ratio of the product between the chemical shift difference between populated states (Δ*ω*) and τ*_ex_* ((Δ*ω·*τ*_ex_*)^−1^) which for slow, intermediate, and fast processes take on the values of < 1, ≈ 1, and > 1, respectively [163]. For processes that may take place in the supra-τ_c_ range (10s of μs and faster) motion is in the fast regime and therefore a given resonance position will represent a population weight of all assumed states. Since conformational exchange is governed by perturbations in *ω*, the line width of an NMR lineshape is affected during a period of free-precession. Assuming a two-state model, the contribution to the line width due to fast exchange is 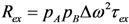 in which *p_A_* is the population of the major state (*p_B_* = 1 – *p_A_*) [159,162]. The next section explores how RD experiments work to disentangle these parameters that modify transverse relaxation.

### 3.2. Relaxation Dispersion Experiments

The two most commonly invoked RD experiments are the transverse-rotating frame relaxation (R_1ρ_) [160,164] and Carr-Purcell-Meiboom-Gill (CPMG) [165,166] experiments. CPMG experiments have grown large popularity to be able to study lowly populated intermediates for some systems [147,157,167]. However, RD can also be used to probe the ground states of a protein as well [115]. Other methods have also been proposed for RD experiments [168,169]. Although, the dependence of conformational exchange differs between the R_1ρ_ and CPMG techniques [1,170], the phenomenological concept is the same. In both cases a relaxation rate is monitored as a function of the rate at which a given magnetization coherence can be refocused. CPMG experiments vary the degree of refocusing by changing 180° pulse repetition rates [171,172,173,174], and R_1ρ_ experiments use radio frequency pulses with varying amplitudes and frequency offsets that effectively constrain (spin-lock) a given magnetization coherence [160]. The amplitude of a given spin-lock pulse is denoted as *ν_RF_*. However, due to technical limitations, CPMG experiments, when performed on ^15^N nuclei, permit the observation of motions with a τ*_ex_* of up to ~150 μs [135]. R_1ρ_ experiments were limited to a time resolution of 40 μs, but now *ν_RF_* values permit the observation of kinetic events up to 25 μs (*vide infra*) which has made this NMR experiment a well suited candidate to access kinetic information from the supra-τ_c_ range.

**Figure 2:**
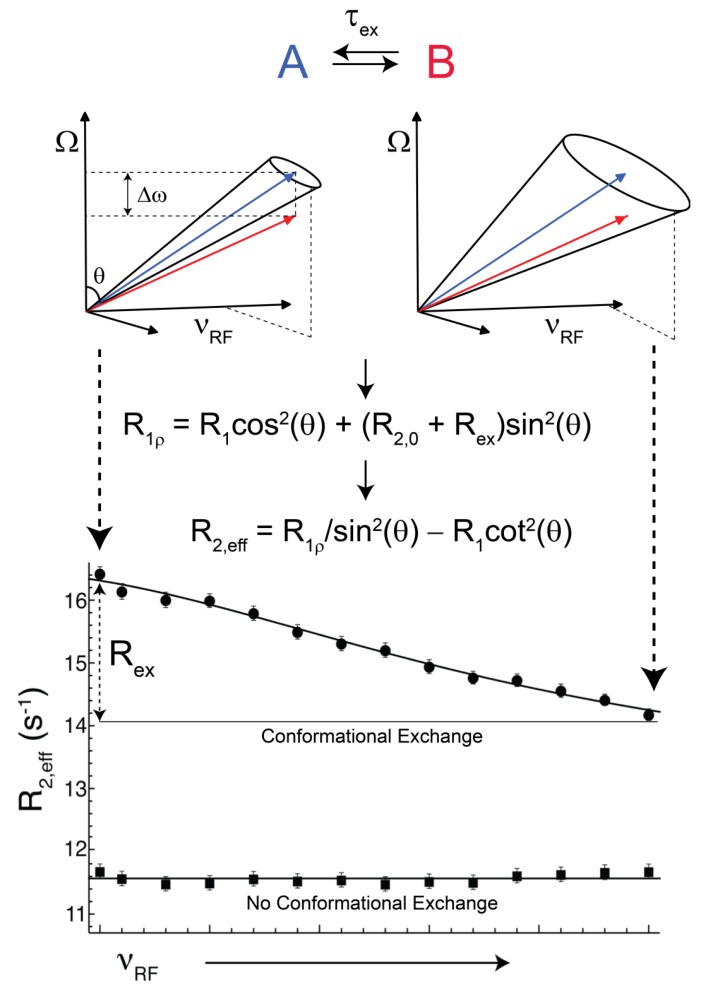
Illustrative schematic describing transverse rotating frame experiments (R_1ρ_) for the measurement of two-state conformational exchange events for NMR active nuclei. As states A and B interconvert with some lifetime (τ*_ex_*) they have a phase separation of Δ*ω*. The length of each vector (arrow tipped lines) denotes the effective field that each populated coherence possesses. The effective field, or length of each vector, is governed by experimental parameters, namely the offset (Ω) and ν*_RF_*, where Ω is the difference between the resonance frequency for a given nucleus and the frequency at which ν*_RF_* is applied. The effective field can be calculated as 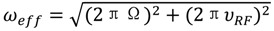 (rad s^−1^). The incomplete refocusing of state B (vector diagram on the left) leads to a dephasing of the magnetization, which translates to a larger relaxation rate. Upon sufficient refocusing of both magnetization vectors (vector diagram on the right) the relaxation rate decreases to R_2,0_. The cones directly reflect the size of the nutation generated from the applied spin-lock field. In the fast regime, the dependence of R_2,eff_ with an increasing ν*_RF_* gives a Lorentzian profile [Equation (44)]. If no conformational exchange exists, then R_2,eff_ remains constant for all applied ν*_RF_* values.

### 3.3. Off/On-Resonance R_1ρ_

Off- and on-resonance R_1ρ_ experiments have been applied to a variety of topics related to the study of internal molecular motions such as hinge and loop displacements [175,176], fast folding events [177], structural configurations that can be assumed in solution by DNA/RNA [178,179], and molecular recognition events [115]. R_1ρ_ experiments can be used as a type of RD experiment because they create “dispersion” by monitoring a nuclei specific relaxation rate as a function of the amplitude of a spin-lock field (*ν_RF_*) and/or by manipulation of the offset frequency (Ω). The transverse rotating frame relaxation rate is dependent on the intrinsic R_1_, R_2,0_, and if conformational exchange exists R_ex_. In the off-resonance R_1ρ_ scenario, populated coherences are rotated away from the static magnetic field by some tilt angle (θ), where the magnetization is locked and thereby begins to precess with the applied field (Figure 2). The tilt angle is an experimentally controlled parameter, θ = tan^−1^(*ν_RF_*/Ω) where *ν_RF_* and Ω are the amplitude of the employed spin-lock (Hz) and the frequency difference between the resonance position of a given nuclei and the frequency at which *ν_RF_* is applied, respectively. The overall magnitude of a spin-lock field is called the effective field (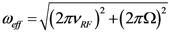
(rad·s^−1^)). If an interconversion event exists, then assuming a two-state process, the populated coherences will be differentially spin-locked (Figure 2). At this point, the alternatively populated coherence is not sufficiently refocused and dephasing leads to an elevated effective relaxation rate (R_2,eff_) (Figure 2). As *ω_eff_* is sufficiently increased to encompass the exchanging magnetization vectors, the relaxation rate decreases to R_2,0_ or to the point at which the exchange contribution to R_2,eff_ is quenched (Figure 2).

The equation that describes off-resonance R_1ρ_ is given in Figure 2. In order to isolate the effect of conformational exchange, it is convenient to visualize the contribution of R_ex_ by either converting off-resonance R_1ρ_ data to R_2,eff_ (Figure 2) or by performing R_1ρ_ experiments on-resonance (θ = π/2) whereby the dependence of R_1_ and the tilt angle are removed. If motion exists in the fast regime, then this creates an addendum to R_1ρ_ that is given by:

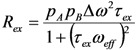
(43)

**Figure 3:**
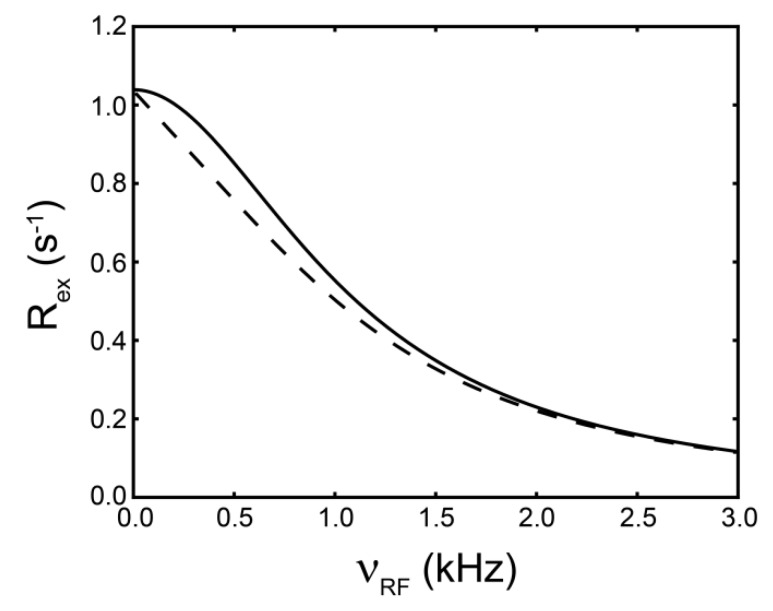
Dependence of R_ex_ monitored by R_1ρ_ (solid black line) and CPMG (dashed black line) experiments. The dashed black line was created using the Carver-Richards model [181] which is applicable for CPMG experiments, and the solid black curve calculated using Equation (44). The exchange parameters *τ_ex_*, *p_B_*, and Δ*ω*, were set to 150 μs, 5 %, and 61 Hz, respectively.

However when the motion is fast, then information between the populations and Δ*ω* cannot be separated and therefore only the product is retained. This is called the conformational amplitude of the process and is denoted as Ф_ex_. Comparing the dependence of R_ex_ between R_1ρ_ and CPMG type experiments it can be seen that their dependence is similar (Figure 3). The 180° pulse repetition rate (*ν_CPMG_*) can be equated to *ν_RF_* using relations derived from Ishima *et al.* (
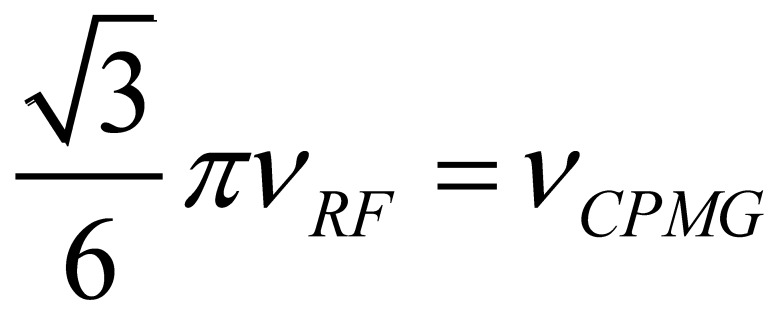
) [180].

It is also important to note that, similar to CPMG experiments, theoretical formalisms and experiments have been reported in which R_1ρ_ can be applied outside of the fast regime [182,183,184]. Still conventional ^15^N CPMG experiments are limited to motions around 150 μs (*ν_CPMG_* ~ 1 kHz) [135]. Taken together, since supra-τ_c_ motion can reside past this limit, R_1ρ_ can be used in hopes of gaining access to the kinetics from this time-scale.

### 3.4. Off-Resonance R_1ρ_ in Super-Cooled Conditions

Kinetics from the supra-τ_c_ range measured by off-resonance R_1ρ_ was recently performed on the protein ubiquitin free in solution [115]. This study characterized a rapid microsecond process within the ground-state ensemble of ubiquitin whose rate of conformer interconversion has implications for its molecular recognition process [115,185,186]. At high temperatures, ubiquitin does not report on having an exchange contribution to R_1ρ_ [70]. The authors hypothesized that if the motion escaped detection at higher temperatures then by lowering the temperature the lifetime of motion would increase making it accessible by off-resonance R_1ρ_. Experiments were performed by super-cooling the sample to 265 K whereby significant RD was detected and a τ*_ex_* of 120 μs was determined [115]. Following the temperature dependence of this motion, an Arrhenius extrapolation identified that the motion at physiological temperatures is between 1 and 19 μs. This motion has been attributed to the interconversion between distinct ubiquitin conformers and was corroborated with predicted Ф_ex_ values from the RDC derived ensembles [28,116], as well as measurements in solution using dielectric relaxation spectroscopy [115,187]. Although dielectric relaxation does not maintain atomic resolution like RD experiments, an Arrhenius extrapolation of the conformer interconversion lifetime is not required. Even with this milestone of measuring RD at super-cooled temperatures, only limited information with respect to the expansiveness of this motion could be attained because only a few sites within ubiquitin were detectable.

### 3.5. Exceeding the Limit with Cryogenically Cooled Probeheads

A prime limitation in RD experiments is the minimum accessible time-scale. Equation (44) has a Lorentzian form and therefore the smallest lifetime that can be observed is limited to the natural line width of a Lorentzian, which is controlled by *ω_eff_* (τ*_ex_* ≈ 1*/ω_eff_*). For the observation of exchange events, *ω_eff_* depends on both *ν_RF_* and Ω. Since these are both experimentally controlled parameters, they can be changed, but a compromise has to be made with respect to the tilt angle. A caveat emerges in which larger Ω values will cause the R_1ρ_ value to be dominated by R_1_ (cos^2^(θ) approaches 1), which in turn minimizes the contribution of R_ex_. Thus, increases in *ν_RF_*, instead of Ω, would provide a means of further extending R_1ρ_ based RD techniques into the supra-τ_c_ range. 

Recently, the previous limitations were exceeded through the use of hardware that is found in many NMR based laboratories, the cryogenically cooled probehead (cryo-probehead). A Bruker QCI S3 cryo-probehead was demonstrated to be capable of safely withstanding *ν_RF_* values up to 6.4 kHz and represents an improvement by a factor of three from what was previously accepted [26]. This larger attainable spin-lock field strength permits a time resolution for motion up to 25 μs. On-resonance R_1ρ_ experiments for ^15^N nuclei within ubiquitin that had been previously shown to have dispersion were performed as validation for the use of large amplitude *ν_RF_* [115,185]. Additional advantages also emerged when a cryo-probehead was used for RD measurements. Since a major aspect in relaxation experiments is to attain sufficient signal to noise that minimizes the errors in the obtained rates, measurement with a helium cooled NMR coil and pre-amplifier fulfills exactly this requirement via a noise reduction in those electronic components. Therefore, R_1ρ_ rates could be monitored with increased precision. Given the increased precision in the measured rates, the errors in the extracted exchange parameters also decreased. More importantly, the implementation of on-resonance experiments removes any contribution from R_1_ and tilt angle to the measured rate. Thus, any observed conformational exchange is solely modulated by *ν_RF_*. The on-resonance RD measurement allows for a more complete sampling of the R_ex_ contribution. Furthermore, a ^15^N site that undergoes smaller amplitude motion within the supra-τ_c_ range can be detected. The use of large amplitude spin-lock field strengths also purported more accurate intrinsic relaxation rates because exchange events up to 25 μs could be removed from R_2,eff_ [26]. The application of more efficient quenching of conformational exchange has been recently demonstrated for the measurement of veracious intrinsic relaxation rates that supplement constant time CPMG type RD experiments [171]. An experiment was created (HEROINE) in which the same averaged coherence is monitored, but large amplitude *ν_RF_* are employed permitting more accurate and precise kinetics to be extracted from CT-CPMG data even when motion is in the fast regime [188] ((τ_ex_·*ω*)^-1^ > 3) The use of large *ν_RF_* may also be extended to nuclei with a larger gyromagnetic ratio (γ) as the achievable *ν_RF_* scales with this value (*ν_RF_* = γ*B_RF_*/2**π**) and even larger *ν_RF_* could then potentially be applied [189,190,191]. Ultimately, large amplitude *ν_RF_* based R_1ρ_ appears to be a promising approach to the further quantification of kinetics from the supra-τ_c_ range.

### 3.6. Experimental Aspects for Kinetic Measurements in the Supra-τ_c_ Range

Experimental outlines for the execution of off/on-resonance R_1ρ_ experiments for ^15^N nuclei have been given in many reviews and texts [132,135,159,160,192], but details pertaining to super-cooled and large amplitude *ν_RF_* measurements will be discussed here. Initially demonstrated by Szyperski and coworkers, super-cooled relaxation measurements can be used to probe sub-τ_c_ events [193,194]. Under super-cooled conditions, the surface tension of the water increases resulting in a lower freezing point for a liquid solution. The NMR sample is initially centrifugated for a sufficient time in order to remove any potential nucleation points for ice formation. The samples can be placed into 1 mm NMR tubes, which are then filled with the liquid samples and subsequently flame sealed. Approximately ten to twelve 1 mm NMR tubes can be placed into a 5 mm tube, which is then inserted in the NMR magnet. Cooling to the desired temperature should be done in small increments and the sample given sufficient time in between temperature decrements for equilibration. As the temperature decreases, the line broadening of the NMR peaks is increased due to an increase in τ_c_ and/or due to slowing down the rates of conformational exchange processes. In order to enhance the sensitivity, R_1ρ_ based RD can be performed by either querying the narrower doublet for ^15^N nuclei [195,196] or by a conventional pulse sequence equipped with a TROSY [197,198] readout to maximize the longevity of the NMR signal. Additionally, TROSY is without decoupling during acquisition and therefore the overall heating canbe reduced.

For the measurement of large amplitude *ν_RF_*, the proper calibration of useable spin-lock field strengths must be ascertained. Continuous wave (CW) off-resonance decoupling experiments are a facile way to determine these amplitudes. CW-fields are applied off-resonance during the acquisition of a two-dimensional correlation experiment (e.g., [1H, 15N]-HSQC) that will cause some amount of partial decoupling. This partial decoupling translates to an effective scalar coupling value which can be correlated with respect to the Ω of a given resonance position [160,192]. A linear correlation between the scalar coupling values and Ω yields a line whose slope is *ν_RF_* [26,160]. The collection of enough points during the acquisition period provides a way to determine the spin-lock amplitudes and lengths that can be safely applied on a given probehead. Of extreme importance is the utilization of recycle delays that are long enough to ensure a duty cycle that does not exceed 5% and that maintain the NMR coil temperature and preamp power reserve at a stable level. Heating due to the electric field component of a radio-frequency pulse can be differential between different experiments. Temperature compensation schemes should be implemented, which not only considers the length that *ν_RF_* is applied, but also the amplitude of *ν_RF_* in order to equalize the temperature between experiments [199]. The relative change in the temperature can be determined from ^1^H^N^ temperature coefficients. 

The acquisition of RD data can be conducted by varying *ν_RF_* and/or Ω. With a given configuration, signal intensities are tracked by measuring their time dependence either by observing a full exponential decay or with two-point sampling schemes. Error estimation and application of different sampling techniques have been previously discussed [135,173,200]. Discerning possible dispersion profiles can be done by following particular selection criteria (i.e. minimum differences in relaxation rates) [147]. From here, the analysis of dispersion data is conducted by standard minimization protocols [132,135], in which the data are fit to models that are with and without conformational exchange contributions. Statistical tests, such as F-tests [201], can be applied in order to identify the better fitting model.

## 4. Conclusions and Outlook

The dynamic characterization of motions from the supra-τ_c_ range has been made possible by the careful dissemination of RDC data collected in unique alignment conditions [102,104,113]. The analysis of such information relies on model dependent (GAF; Section 2.4) [109,110,111] and model independent techniques (MFA, DIDC, SCRM and ORIUM; Section 2.3) [68,72,101,102,104,105]. Insight from such data has highlighted molecular motions related to molecular recognition for ubiquitin [28] and TAR-RNA [202], which were only realized with the inclusion of RDC data. RD is also emerging as an experimental tool to capture the kinetics from the supra-τ_c_ range [26,115]. Further methodological advancement will be required to try to completely sample this four orders of magnitude time window, however motion as fast as ~25 μs can be accessed with atomic resolution [26] based on variance of ^15^N chemical shifts and even faster for nuclei with a larger gyromagnetic ratio. The harmony between experiment and computer simulation can also aid in accelerating studies of protein dynamics from supra-τ_c_ range. The RDC-derived ensembles [28,115,116] have shown that they faithfully include structural variances within the supra-τ_c_ range, and can also be used to have predictive power in identifying sites that may undergo conformational exchange that could be detectable by RD. Additionally, long MD trajectories and AMD type simulations have been able to identify supra-τ_c_ motion for the backbone of BPTI [20] and for example thrombin [120,121], respectively. A major goal for the future will be to see these experimental and computational techniques applied to an increased number of other biological macromolecules in order to enhance our understanding of the complex behavior displayed by biological macromolecules within the supra-τ_c_ window.
